# Fitness Costs Predict Inbreeding Aversion Irrespective of Self-Involvement: Support for Hypotheses Derived from Evolutionary Theory

**DOI:** 10.1371/journal.pone.0050613

**Published:** 2012-11-28

**Authors:** Jan Antfolk, Debra Lieberman, Pekka Santtila

**Affiliations:** 1 Department of Psychology and Logopedics, Abo Akademi University, Turku, Finland; 2 Department of Psychology, University of Miami, Coral Gables, Florida, United States of America; University of Sheffield, United Kingdom

## Abstract

It is expected that in humans, the lowered fitness of inbred offspring has produced a sexual aversion between close relatives. Generally, the strength of this aversion depends on the degree of relatedness between two individuals, with closer relatives inciting greater aversion than more distant relatives. Individuals are also expected to oppose acts of inbreeding that do not include the self, as inbreeding between two individuals posits fitness costs not only to the individuals involved in the sexual act, but also to their biological relatives. Thus, the strength of inbreeding aversion should be predicted by the fitness costs an inbred child posits to a given individual, irrespective of this individual’s actual involvement in the sexual act. To test this prediction, we obtained information about the family structures of 663 participants, who reported the number of same-sex siblings, opposite-sex siblings, opposite-sex half siblings and opposite-sex cousins. Each participant was presented with three different types of inbreeding scenarios: 1) Participant descriptions, in which participants themselves were described as having sex with an actual opposite-sex relative (sibling, half sibling, or cousin); 2) Related third-party descriptions, in which participants’ actual same-sex siblings were described as having sex with their actual opposite-sex relatives; 3) Unrelated third-party descriptions, in which individuals of the same sex as the participants but unrelated to them were described as having sex with opposite-sex relatives. Participants rated each description on the strength of sexual aversion (i.e., disgust-reaction). We found that unrelated third-party descriptions elicited less disgust than related third-party and participant descriptions. Related third-party and participant descriptions elicited similar levels of disgust suggesting that the strength of inbreeding aversion is predicted by inclusive fitness costs. Further, in the related and unrelated conditions alike, the strength of inbreeding aversion was positively associated with the degree of relatedness between those described in the descriptions.

## Introduction

Offspring born to first-degree relatives are 17%–40% more likely to suffer disease and death as compared to children born to non-relatives [1]–[3]. The lowered biological fitness of inbred offspring, referred to as inbreeding depression, has been explained as being due to the increased likelihood of detrimental homozygous recessive allele combinations and the increased susceptibility to disease-causing organisms [4], [5]. Inbreeding depression suggests a strong selective pressure against the selection of close genetic relatives as sexual partners–in humans and non-human species alike. While the long-enduring evolutionary effects of inbreeding depression are expected to have produced proximate psychological mechanisms that generate sexual aversion toward close relatives, the strength of the aversion should depend on the type of relative–the lower the coefficient of genetic relatedness, the weaker the felt aversion. For example, the relative biological cost of inbreeding to a given individual is twice as high when inbreeding with a full sibling as compared to a half sibling.

The costs of inbreeding, however, extend beyond the two related individuals involved in the sexual union. This is because an individual’s reproductive success is not limited to the number or quality of offspring produced directly. Instead, reproductive success is measured by the total number of allele copies that an individual is able to transmit further due to their own (in)actions, either through direct descendants or through offspring of related individuals such as the individual’s siblings or cousins. This logic is captured by inclusive-fitness theory [6]. Inclusive-fitness theory suggests that alleles resulting in aiding kin can spread in the population. The implications of this perspective on kin-directed altruism and inbreeding aversion are profound: Inclusive-fitness theory, for example, clarifies why we are motivated to be altruistic toward kin other than our own offspring. Because siblings and cousins (and their offspring) have an increased probability of sharing copies of the alleles underlying altruistic behaviors, investing in their well-being and reproductive success furthers the likelihood that these alleles will be transmitted down the generations. Conversely, alleles contributing to *not* acting in a manner that is harmful to close kin can also spread in the population following the same principle. Thus, by harming the reproductive success of our kin, we indirectly harm our own fitness, and alleles counteracting such behaviors should be selected for. In the case of inbreeding, this implies that inbreeding between two individuals (e.g., a father and sister) bears fitness costs to their genetic relatives (e.g., the sister’s siblings), although they themselves do not participate. Consider the position of a relative with the opportunity to intervene in or prevent incestuous mating. In the case of father-daughter incest, by not acting, an uninvolved brother or sister bears the cost of losing a potentially healthy niece/nephew of r = 1/4 (had the daughter had a child with an unrelated male) for an inbred sibling-niece/nephew with an r = 3/4, but suffering from inbreeding depression. Whether this pays off in fitness terms depends on the magnitude of the inbreeding depression and the certainties of relatedness between the individuals involved. These extended costs of inbreeding and the conflicts that ensue have been modeled by Haig [7]. Importantly–and our focus here–Haig’s model suggests that psychological mechanisms that oppose inbreeding should be sensitive not only to the costs of having sex with a relative *oneself*, but also to the costs imposed by *two other* relatives engaging in sex. In general, the closer the relatedness of the incestuous relatives to each other and to oneself, the greater the costs of inbreeding, and the greater the opposition to such acts.

To further consider the costs of inbreeding, one also has to consider the asymmetry in parental investment between the sexes. Women have a minimum investment of nine months of gestation and an additional period of lactation during which fertility is usually suppressed, whereas men’s minimum investment is nothing more than the duration of intercourse. Thus, the obligatory parental investment in offspring is higher for women than for men [8]. Measured as a lost opportunity to invest in more optimal offspring, the individual costs of engaging in inbreeding are larger for women than for men. Due to this difference in opportunity costs, it is expected that women should be more aversive to inbreeding than men. Using reactions to descriptions of unrelated individuals engaging in inbreeding studies have confirmed this sex difference [9], [10].

### The Present Study

Although evolutionary theory suggests clear-cut predictions concerning factors that should influence the intensity of inbreeding aversion, the empirical literature is currently relatively limited. Under the assumption that aversive reactions to inbreeding are an adaptive response developed over time to down-regulate willingness to engage in sexual activity with family members, it is expected that the strength of this aversion should be positively associated with the cost to a given individual. So far, the most extensively researched topic is the effect of environmental cues of relatedness on the strength of inbreeding aversions. This has been addressed in anthropological studies [11]–[14], self-reports of actual sexual behaviors [15], [16], and by using reactions to descriptions of individuals that are not related to the participant engaging in inbreeding (i.e. third-party descriptions) [10], [17]. In studies using reactions to third-person inbreeding descriptions, it has been shown that the cues thought to govern the development of personal sexual aversions toward one’s own siblings (e.g., childhood coresidence duration and seeing a younger sibling being nursed by a person identified as one’s own mother) also influence reactions to third-person sibling incest [17]–[19]. These studies have generally confirmed predictions derived from evolutionary theory.

However, there is a gap in the empirical literature investigating the relationship between degree of relatedness and the strength of inbreeding aversion. To our best knowledge no study has been designed to test this in a robust and comprehensive fashion. One of two studies that pertain to this issue was carried out by Quinsey, Lalumière, Querée, and McNaughton [20] who investigated how reactions to intrafamilial sexual activity varied depending on the degree of relatedness between the participants. The authors found a positive association between degree of relatedness and severity of reactions towards the sexual activity. Another study, by Antfolk, Karlsson, Bäckström, and Santtila [9] showed that degree of relatedness (biological vs. non-biological) moderated disgust elicited by descriptions of sexual activity between family members, such that biological incestuous sexual activity was found more disgusting than sociolegal incestuous sexual activity. In both studies only third-person descriptions were used to measure the reactions towards inbreeding, making it impossible to interpret them as evidence for inclusive fitness.

To more rigorously test predictions derived from inclusive-fitness theory, we designed a web-administered study in which we obtained information about each participant’s individual family structure. Participants were asked to report the number of same-sex siblings, opposite-sex siblings, opposite-sex half siblings and opposite-sex cousins. Each participant was subsequently presented with three different types of inbreeding descriptions: Participant inbreeding descriptions, in which the participants themselves were described as having sex with their actual opposite-sex relatives (sibling, half sibling, or cousin); related third-party inbreeding descriptions, in which the participants’ actual same-sex siblings was described as having sex with the participants’ actual opposite-sex relatives; unrelated third-party inbreeding descriptions, in which individuals of the same sex as the participants but unrelated to them were described as having sex with opposite-sex relatives. In unrelated third-party inbreeding description, unlike in participant and related third-party inbreeding descriptions, there are no fitness costs to the participant. Table 1 shows the description types that were included in the survey (for brevity, we only report descriptions for male participants).

**Table 1:** Possible Inbreeding Descriptions for a Male Participant.

Person *A*	Person *B*	Inbreeding Depression^a^	Inclusive Cost to Participant^b^	
		*r_AB_*	*pr_A_*	*pr_B_*
Participant Inbreeding Descriptions				
Male Participant	Sister	.5	1.0	.5
Male Participant	Half-Sister	.25	1.0	.25
Male Participant	Female Cousin	.125	1.0	.125
Related Third-Party Inbreeding Descriptions				
Male Participant’s Brother	Sister	.5	.5	.5
Male Participant’s Brother	Half-Sister	.25	.5	.25
Male Participant’s Brother	Female Cousin	.125	.5	.125
Unrelated Third-Party Inbreeding Descriptions				
Unrelated Male	Their Sister	.5	.0	.0
Unrelated Male	Their Half-Sister	.25	.0	.0
Unrelated Male	Their Female Cousin	.125	.0	.0

Note: ^a^Inbreeding Depression describes the relative fitness decrease in the inbred offspring that is a direct function of the degree relatedness of those described as participating in inbreeding (i.e. Person A and Person B). ^b^Inclusive Cost to Participant describes the cost to the participant that is a function the degree of relatedness of the participant to both persons described as participating in inbreeding. (i.e. Person A and Person B).

### Disgust as a Measure of Aversion

Westermarck [21] suggested that inbreeding aversion is mediated via negative emotions associated with the idea of engaging in sexual activity with one's close relative. Indeed, the feeling of disgust has been shown to be negatively associated with sexual arousal as a response to erotic films [22], [23] and to erotic stories [24]. Similarly, disgust has a negative influence on the willingness to engage in a number of sexual behaviors [25] including sexual behavior with close kin [26]. Third-person descriptions of inbreeding have also been found to elicit disgust [10], [27], [28] rather than other negative emotions or reactions, such as fear, sadness, shame, confusion, or guilt [29]. Interestingly, disgust was also the most commonly described emotional reaction to experienced inbreeding in a large-scale victimization study of a Finnish population sample [30]. Following this evidence, we operationalized inbreeding aversion as the feeling of disgust elicited by descriptions of sexual relations between close genetic relatives.

### Predictions

1. Based on parental investment theory and earlier findings reviewed above, we predict that women would report stronger aversive reactions to all descriptions of inbreeding than men would.
2. Because engaging in inbreeding oneself and instances of inbreeding between one’s close relatives impose greater fitness costs as compared to instances of inbreeding between unrelated third parties, we predict that participant inbreeding descriptions and related third-party inbreeding descriptions would elicit stronger aversive reactions than unrelated third-party inbreeding descriptions.
3. Likewise, because engaging in inbreeding oneself imposes higher fitness costs to the participant than inbreeding between persons related to the participant, we predict that participant inbreeding descriptions should be found more aversive than related third-party inbreeding descriptions.
4. As degree of relatedness between those engaging in inbreeding is positively associated with fitness costs for both men and women, we predict that for both sexes the degree of relatedness between those engaging inbreeding in the participant and the related third-party descriptions would moderate disgust reactions such that the greater the degree of relatedness, the greater the disgust reported.
5. Given previous findings showing that the strength of personal sexual aversions toward engaging in incest oneself shapes attitudes regarding third party incest [18], we predicted that for the unrelated third-party inbreeding descriptions, the closer the degree of relatedness between the individuals described, the greater the reported disgust.

With these predictions in mind, we developed the following study.

## Methods

### Participants

The sample consisted of 663 (475 women and 188 men) graduate and post-graduate students at Abo Akademi University in Turku, Finland. We sent an invitation to an e-mail list containing addresses to currently enrolled graduate and post-graduate students at Abo Akademi University to participate in the study in April 2011. A reminder was sent one week later. Both e-mails contained brief information of the survey, an assurance of participants’ anonymity, as well as a link to a web site containing the experiment. Participation in an optional lottery of a 200€ gift-card to a travel bureau was offered. To ensure anonymity, information to participate in the lottery was collected through a separate web site. Participants were informed about the sensitive content of the study, informed about the measures taken to ensure anonymity (i.e., by not registering any information such as IP-addresses or names that could be linked to specific individuals), and informed that they could stop the survey at any point. Only data provided from completed survey were used in the analyses. The present study was approved by the Institutional Review Board at the Department of Psychology and Logopedics at Abo Akademi University.

### Procedure

Participants were asked to report the number of same-sex siblings, opposite-sex siblings, opposite-sex half siblings via the mother, opposite-sex half siblings via the father, and opposite-sex cousins via both matrilineal and patrilineal aunts and uncles. If a participant reported having more than one such relationship in any of these categories, then one of these individuals was randomly selected for subsequent questioning. In order to facilitate subsequent information gathering, the respondent was asked to provide the name of each of these randomly selected relatives. The names were not saved in the data file and this procedure therefore did not endanger anonymity.

Each participant was presented with three different types of inbreeding descriptions: participant inbreeding descriptions, in which the participants themselves were described as having sex with their actual opposite-sex relatives (sibling, half sibling, or cousin), for example “you having sex with your sister Jane”; related third-party inbreeding descriptions, in which the participants’ actual same-sex sibling was described as having sex with the participants’ opposite-sex relatives, for example “your brother John having sex with your sister Jane”; unrelated third-party inbreeding descriptions, in which a same-sex individual unrelated to the participant was described as having sex with opposite-sex relatives, for example “a man having sex his full sister”. The number of participant and related third-party inbreeding descriptions was determined by the number of relationships reported by each participant. For all participants unrelated third-party inbreeding descriptions included all possible relationships. For each inbreeding description the participants were asked to self-report their level of disgust on a Likert-type scale with the anchors 0 (not at all disgusting) and 9 (extremely disgusting).

As there were three different types of inbreeding descriptions (participant, related third-party, and unrelated third-party) and up to nine different levels of relatedness between those described as participating in inbreeding (the participants themselves, opposite-sex siblings, opposite-sex half siblings via the mother, opposite-sex half siblings via the mother, and opposite-sex cousins via both matrilineal and patrilineal aunts and uncles) we wanted to control for possible order effects. Because the software used for administering the web-administered experiment did not allow for a full randomization, we created three different experiment versions counterbalancing the three different types of inbreeding descriptions using a Latin Square procedure and pseudo randomizing the levels of relatedness between those described as participating in inbreeding, setting a randomized order for each different level of inbreeding description type and experiment version. Thus, the eventual order effects were counterbalanced across the whole study. In all, three different versions of the web-administered experiment were created for each sex yielding a total of six experiment versions. Participants were asked to choose a version according to their month of birth, distributing the participants evenly across the different versions.

### Statistical Analyses

For all statistical analyses we used SPSS 19. As we expected observations within each individual to be correlated, we analyzed data using Generalized Estimation Equations, which fit a generalized linear model to observations with an unknown correlation structure [31]. The dependent variable disgust was somewhat positively skewed, and so for this reason we replicated each analysis using a logarithmic transformation of the variable. Results with the transformed variable did not differ from the original variable and thus we report results using the original scale. Last, due to the low number of observations, we collapsed maternal and paternal half siblings into one category labeled half siblings, and maternal and paternal cousins into one category labeled cousins.

## Results

### Descriptive Results

Women in our sample were younger than the men (*M*_women_ = 25.3, *SD* = 6.8 and *M*_men_ = 27.8, *SD* = 7.7, *t*[661] = 4.20, *p*<.001). For siblings, 66.1% of the participants reported one or more same-sex siblings, 60.1% reported one or more opposite-sex siblings, 5.8% reported one or more opposite-sex half siblings via their mother, and 6.8% via their father. For matrilineal cousins, 59.2% of the participants reported one or more opposite-sex cousins via an aunt, and 66.3% via an uncle. For patrilineal cousins, 52.1% of the participants reported one or more opposite-sex cousins via an aunt and 48.1% via an uncle.

### The Effect of Sex on Disgust Reactions to Inbreeding Descriptions

We expected that women would be more disgusted by inbreeding than men due to women having a relatively increased opportunity cost associated with sub-optimal offspring. Indeed, we found a significant effect of sex on disgust reactions to inbreeding descriptions overall (Wald χ^2^(1) = 32.23, *p*<.001) with women (*M* = 8.04, *SE* = 0.54) being more disgusted than men (*M* = 7.21, *SE* = 1.36). We then replicated this finding across all three types of inbreeding descriptions (all *p*s<.001), and across all three degrees of relatedness between those described as participating in inbreeding descriptions (all *p*s<.001).

### The Effect of the Type of Inbreeding Description on Disgust Reactions

**Figure 1:**
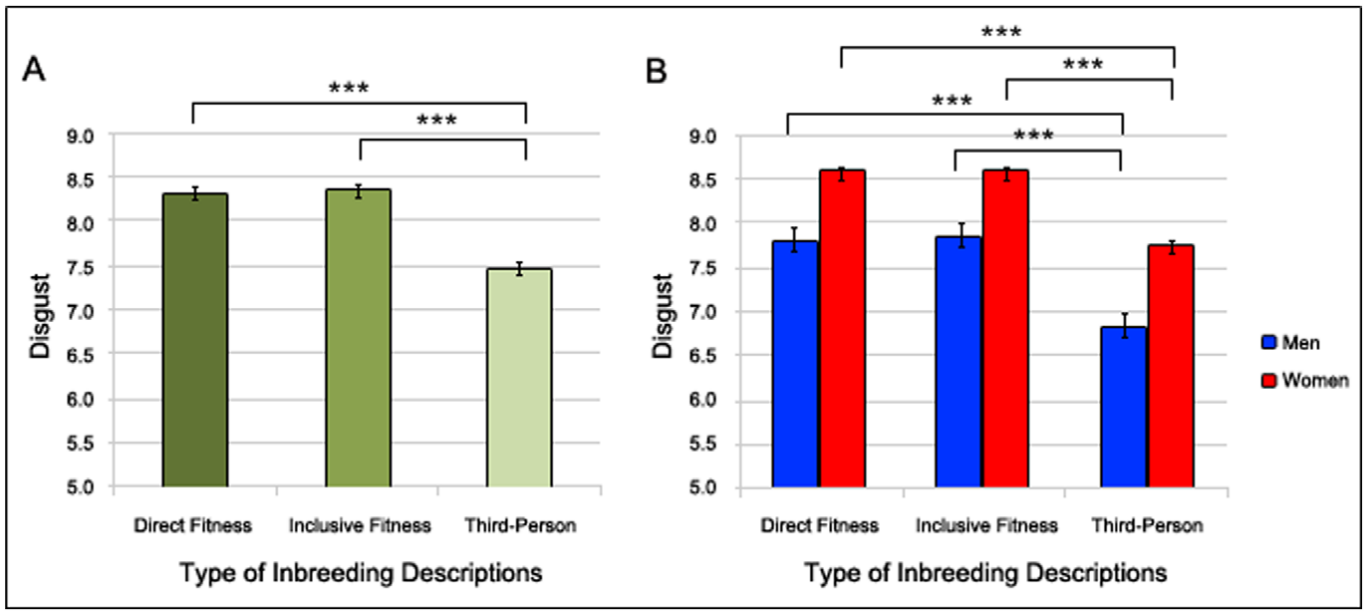
The Effect of Types of Inbreeding Descriptions on Inbreeding Aversion. The effect of types of inbreeding descriptions (participant inbreeding descriptions, in which the participants themselves were presented as having sex with their actual opposite-sex relatives; related third-party inbreeding descriptions, in which the participants’ actual same-sex sibling was presented as having sex with the participants’ opposite-sex relatives; unrelated third-party inbreeding descriptions, in which a same-sex individual unrelated to the participant was presented as having sex with their opposite-sex relatives) on self-reported disgust with higher values indicating stronger disgust reactions. The left panel A consider men and women simultaneously and the right panel B men and women separately. † *p*>.1 * *p*>.05, ** *p*>.01, *** *p*>.001.

We expected that participant inbreeding descriptions and related third-party inbreeding descriptions would elicit stronger aversive reactions than unrelated third-party inbreeding descriptions, and furthermore, that participant inbreeding descriptions would be found more aversive than related third-party inbreeding descriptions. We found a significant effect of type of inbreeding description on levels of elicited disgust (Wald χ^2^(2) = 191.15, *p*<.001). A planned comparison revealed that participants reported significantly less disgust to unrelated third-party inbreeding descriptions than to participant (*p*<.001) and related third-party (*p*<.001) inbreeding descriptions. However, there was no significant difference between participant inbreeding descriptions and related third-party inbreeding descriptions (*p*>.05). See Figure 1 for means and standard errors.

Next, we recalculated these analyses separately for men and women to see whether the differences were the same in spite of the general level difference in disgust between men and women. We found the same significant effect for both women (Wald χ^2^(2) = 149.05, *p*<.001) and men (Wald χ^2^(2) = 52.87, *p*<.001). Planned comparisons revealed that participants of both sexes reported significantly less disgust to unrelated third-party inbreeding descriptions than to participant (*p*<.001 for both women and men) and related third-party (*p*<.001 for both women and men) inbreeding descriptions. However, there was no significant difference between participant and related third-party inbreeding descriptions for either sex (*p*>.05 for both women and men). See Figure 1 for means and standard errors.

### The Effect of Relatedness between Those Described as Participating in Inbreeding Descriptions on Disgust Reactions

**Figure 2:**
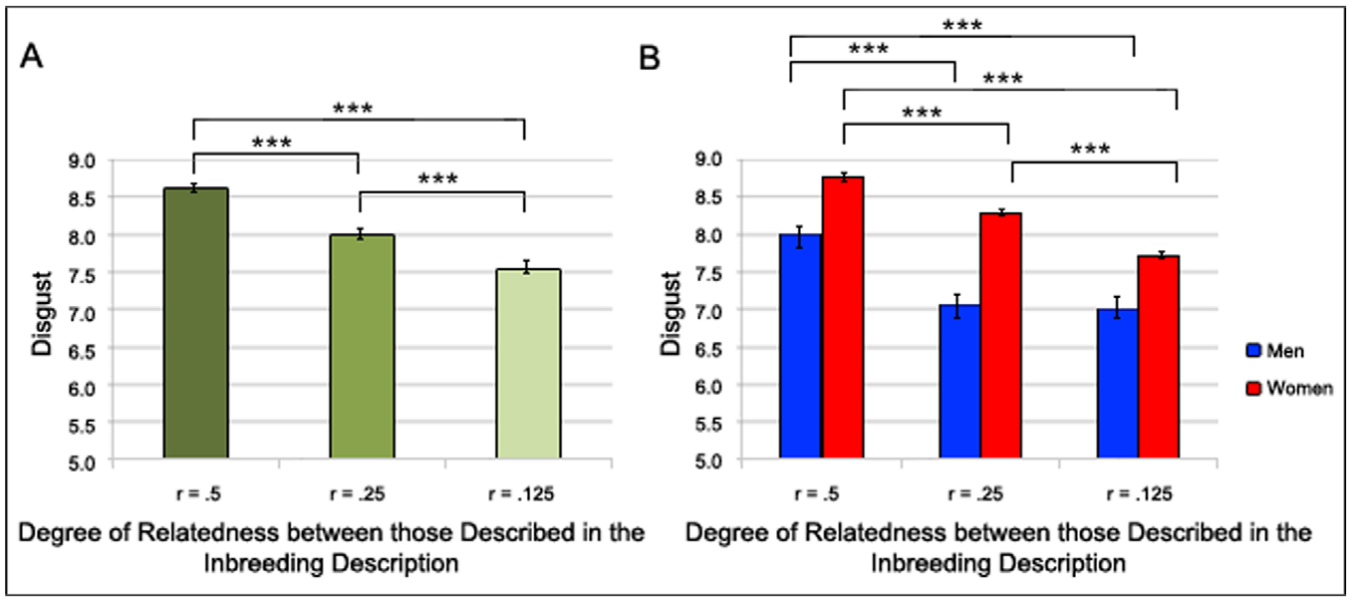
The Effect of Degree of Relatedness on Inbreeding Aversion. The effect of degree of relatedness (*r* = .50 [full siblings], *r* = .25 [half siblings], and *r* = .125 [cousins]) between those described as participating in inbreeding descriptions on self-reported disgust with higher values indicating stronger disgust reactions. The left panel A considers men and women together and the right panel B considers men and women separately. † *p*>.1 * *p*>.05, ** *p*>.01, *** *p*>.001.

We expected that the degree of relatedness between those described as participating in the inbreeding descriptions would moderate disgust reactions such that the higher the relatedness the more disgust the descriptions would elicit. Indeed, we found a significant effect of relatedness between those described as participating in inbreeding (*r* = .50, *r* = .25, and *r* = .125) on levels of elicited disgust (Wald χ^2^(2) = 356.06, *p*<.001). A planned comparison showed that all comparisons were significant (all *p*s<.001). See Figure 2 for means and standard errors.

Again, we recalculated this analysis separately for men and women to check that the effects would be the same in the two groups. We found that this was the case for both women (Wald χ^2^(2) = 282.05, *p*<.001) and men (Wald χ^2^(2) = 92.58, *p*<.001). Pair-wise comparisons revealed that for women all comparisons were significant (all *p*s<.001). For men, however, we found no difference between inbreeding between cousins and between half-siblings (*p*>.05). Other comparisons were significant (*p*s<.001). See Figure 2 for means and standard errors.

Examining each inbreeding description separately, we found that the degree of relatedness between the sexual partners moderated disgust in both the participant inbreeding descriptions (Wald χ^2^(2) = 60.02, *p*<.001) and the related third-party inbreeding descriptions (Wald χ^2^(2) = 40.53, *p*<.001). Next, we conducted planned analyses of the effect of degree of relatedness within each type of inbreeding description. In participant inbreeding descriptions both *r* = .50 and *r* = .25 significantly differed from *r* = .125 (both *p*s<.001). There was, however, no significant difference between *r* = .50 and *r* = .25 (*p*>.05). In related third-party inbreeding descriptions *r* = .50 significantly differed from *r* = .125 (*p*<.001). Inbreeding descriptions with a relatedness of *r* = .25 between those described did not differ significantly from the others (both *p*s*>*.05).

**Figure 3:**
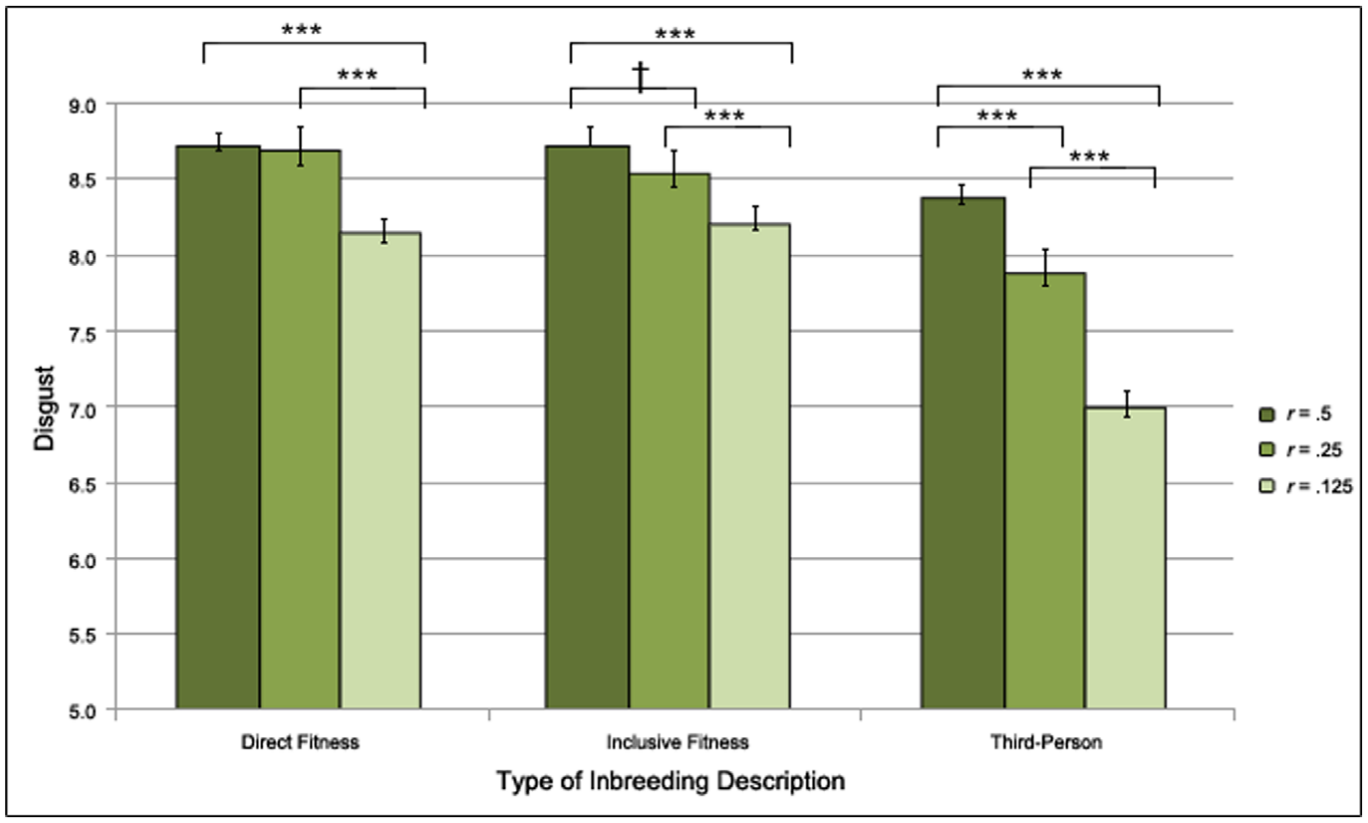
The Effect of Relatedness within Inbreeding Descriptions on Inbreeding Aversion. The effect of relatedness (*r* = .50 [full siblings], *r* = .25 [half siblings], and *r* = .125 [cousins]) between those described as participating on self-reported disgust with higher values indicating stronger disgust reactions within each type of inbreeding description; (participant inbreeding descriptions, in which the participants themselves were presented as having sex with their actual opposite-sex relatives; related third-party inbreeding descriptions, in which the participants’ actual same-sex sibling was presented as having sex with the participants’ opposite-sex relatives; unrelated third-party inbreeding descriptions, in which a same-sex individual unrelated to the participant was presented as having sex with their opposite-sex relatives. † *p*>.1 * *p*>.05, ** *p*>.01, *** *p*>.001.

Finally, as earlier studies have shown an effect of degree of relatedness in reactions to third-person inbreeding descriptions, we expected to find an effect of relatedness between those described as participating in these types of inbreeding descriptions. Indeed, in unrelated third-party inbreeding descriptions, we found that degree of relatedness moderated disgust (Wald χ^2^(2) = 342.18, *p*<.001) and that all possible comparisons were significant (all *p*s<.001). See figure 3 for means and standard errors.

## Discussion

We studied the reactions of 663 Finnish students and post-graduate students to various descriptions of inbreeding and provide the first extensive test of the association between the strength of inbreeding aversions and associated inclusive fitness costs. Basing our predictions on inclusive-fitness theory [6], we predicted that the strength of inbreeding aversion would be associated with the fitness costs associated with a particular inbreeding situation such that an individual’s inbreeding aversion would both depend on the relatedness between those engaged in inbreeding and their relationship to the individual. To test these predictions we created a web-administered experiment in which we used the actual relationships present in each participant’s family structure to create descriptions that investigated disgust reactions toward inbreeding descriptions involving the participant directly, the participant’s same sex sibling, and third-parties.

We found that descriptions of participant inbreeding descriptions and related third-party inbreeding descriptions elicited significantly more disgust than unrelated third-party inbreeding descriptions. This suggests that felt disgust is stronger in situations associated with large fitness costs. The difference between levels of disgust reported across these descriptions cannot be parsimoniously explained by the social taboo against incestuous sexual relations as this should have led to an equal level of disgust across all conditions. That is, if external taboos shape personal attitudes, then we should have observed more uniform responses. However, it is plausible that the difference we found is a consequence of an individual being more empathic with their relatives than strangers. Future studies can address this possibility. It should be noted, that the inbreeding aversion elicited by unrelated third-party inbreeding descriptions cannot be parsimoniously explained from fitness costs to a participant. One explanation is that this social extension is a byproduct of the personal inbreeding aversion [17], [21]. That is, when asked to judge an incestuous act between unrelated third parties, one accesses the fitness consequences of engaging in the behavior oneself and generates an attitude accordingly. This could explain why we found that disgust reactions toward unrelated third-party inbreeding was sensitive to the degree of relatedness between those engaging in the sexual act. This is the same pattern we observed for cases involving the participant and the participant’s sibling and is in line with earlier research [8]. For instance, past research has demonstrated that the same kinship cues that predict personal sexual aversions toward one’s own close genetic relatives also predict moral judgments regarding incest between unrelated third parties [18], [19]. Certainties of personal relatedness thus appear to shape attitudes regarding third parties. Recent theorizing explains why this might be so – that is, why we access personal fitness outcomes when judging third party sexual behavior [32], [33].

That we did not find the expected difference between participant inbreeding descriptions and related third-party inbreeding descriptions might be due to several reasons. First, this could be due to the limited variation in the upper end of the scale measuring disgust. This issue could be possibly rectified with an increased sample-size, which would allow for a more powerful statistical analysis of the existing variance. Second, this could be due to both situations being found very aversive and disgusting, not allowing the participants to be able to distinguish between them on the scale we employed. Third, it could be that there is no difference between participant inbreeding descriptions and related third-party inbreeding descriptions, falsifying our prediction. However, the specificity of the other results we obtained makes this interpretation unlikely.

We expected that the degree of relatedness between those described as participating in the inbreeding descriptions would moderate disgust elicited by inbreeding descriptions such that the higher the relatedness the more disgust the descriptions would elicit. We found that the degree of relatedness between those described as participating in the inbreeding descriptions moderated disgust reactions such that the more closely related, the more disgust the inbreeding description elicited in the participants. However, we found no significant difference between sibling inbreeding and half-sibling inbreeding descriptions in participant inbreeding descriptions and only a trend in related third-party descriptions inbreeding descriptions. This may be due to the cell sizes being relatively small in half-sibling inbreeding descriptions, as only 5.8% participants reported one or more opposite-sex half siblings via their mother, and 6.8% via their father. Another explanation is that the environmental circumstances, such as co-residence duration, may not differ very much between half-siblings and full siblings in this sample. As inbreeding aversion has been shown to rely heavily on such environmental cues [10], [17]–[19], future studies should aim to address this possible explanation.

It is important to point out that although the costs of inbreeding may be modeled with precision, the actual inbreeding aversion is not necessarily as precise. There are a number of reasons for this. Rather than being a function of degree of relatedness to a given individual, the intensity of the inbreeding aversion is expected to depend on environmental information correlated with relatedness, such as childhood coresidence [21] or seeing a child nursed by an individual identified as one’s own mother [18]. Although this environmental information is likely correlated with degree of relatedness, it is unlikely a one-to-one match. After all, we can seldom be 100% certain about how closely related we are to another individual. Further, for an inbreeding aversion to evolve, it does not have to operate perfectly. It only needs to operate well enough for the underlying alleles to be more selected than competing alleles. Finally, it is possible that rather than measuring inbreeding aversion, the results may be a function of empathic emotions to the persons described in the inbreeding descriptions. Future studies should aim at addressing this possibility.

There are some limitations to the present study that should be considered. First, the positively skewed distribution and the generally high levels of disgust reported may have decreased variability in the upper end of the scale leading to conservative estimates of level differences. This should perhaps be taken into account when interpreting the non-significant difference between participant inbreeding descriptions and related third-party inbreeding descriptions. In order to avoid this, using different methods for measuring inbreeding aversion should be considered. Second, one concern in the measurement of emotion is that it relies on self-report as studies suggest that subjective and physiological indices of disgust are not always correlated [34].

In order to gain more comprehensible knowledge, future studies should investigate inbreeding aversion including parent-child and other vertical inbreeding instances. While a number of studies have investigated inbreeding aversion between siblings and cousins, there is a lack of theoretically and methodologically sound studies aimed at gaining more understanding of the factors regulating parent-child inbreeding. Moreover, using this method of varying the individuals described as participating in inbreeding, the effects of proximal mechanisms, such as co-residence and phenotypical resemblance could be included to gather a wider understanding of the social extension of the inbreeding aversion. Moreover, the method used in the present study is not limited only to measuring inbreeding aversion. The method could also be used to investigate the specificity of self-regulatory adaptive psychology. For example, this method would allow for testing whether individual variations in fertility across the menstrual cycle only regulates the individual propensity to engage in a given behavior (e.g. risky sexual behavior) or whether it also regulates the reactions to other individuals engaging in this behavior.

This study, with some few exceptions, show that the strength of inbreeding aversion, measured as reactions of disgust to various inbreeding descriptions, is positively associated with the decrease in fitness stemming from a given inbreeding situation. In other words, the strength of inbreeding aversion follows predictions derived from inclusive-fitness theory.
